# MicroRNA‐based recombinant AAV vector assembly improves efficiency of suicide gene transfer in a murine model of lymphoma

**DOI:** 10.1002/cam4.2935

**Published:** 2020-02-28

**Authors:** Nusrat Khan, Sabna Cheemadan, Himanshi Saxena, Sridhar Bammidi, Giridhara R. Jayandharan

**Affiliations:** ^1^ Department of Biological Sciences and Bioengineering Indian Institute of Technology Kanpur UP India; ^2^ Centre for Stem Cell Research Christian Medical College Vellore TN India; ^3^ Department of Hematology Christian Medical College Vellore TN India

**Keywords:** AAV, lymphoma, microRNA, suicide gene

## Abstract

Recent success in clinical trials with recombinant Adeno‐associated virus (AAV)‐based gene therapy has redirected efforts in optimizing AAV assembly and production, to improve its potency. We reasoned that inclusion of a small RNA during vector assembly, which specifically alters the phosphorylation status of the packaging cells may be beneficial. We thus employed microRNAs (miR‐431, miR‐636) identified by their ability to bind AAV genome and also dysregulate Mitogen‐activated protein kinase (MAPK) signaling during vector production, by a global transcriptome study in producer cells. A modified vector assembly protocol incorporating a plasmid encoding these microRNAs was developed. AAV2 vectors packaged in the presence of microRNA demonstrated an improved gene transfer potency by 3.7‐fold, in vitro. Furthermore, AAV6 serotype vectors encoding an inducible caspase 9 suicide gene, packaged in the presence of miR‐636, showed a significant tumor regression (~2.2‐fold, *P* < .01) in a syngeneic murine model of T‐cell lymphoma. Taken together, we have demonstrated a simple but effective microRNA‐based approach to improve the assembly and potency of suicide gene therapy with AAV vectors.

## Graphical abstract

We have developed a microRNA based AAV packaging method. This strategy when tested for its efficacy in vivo in a tumour model of lymphoma in mice enhanced the therapeutic effect of suicide gene transfer.

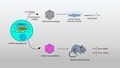

## 1 INTRODUCTION

The broad tissue tropism together with the excellent safety profile has established Adeno‐associated virus (AAV)‐based vectors as the platform of choice for in vivo gene therapy.^1^, ^2^, ^3^ AAV vectors have been translated into the clinic for a variety of conditions, including coagulation disorders, congenital blindness, and neurodegenerative diseases.^4^, ^5^, ^6^, ^7^ Data generated from these clinical studies have also identified factors such as the host immune response and the suboptimal transduction in the target tissue as major barriers for the universal application of AAV vectors in gene therapy. In addition, the high cost of treatment with the current gene therapy drugs,^8^, ^9^, ^10^ pose a significant challenge to its expanded use. These factors underscore the need to generate AAV vectors at significantly higher titers or develop vectors with enhanced potency with the existing vector production methods. This in‐principle should allow for enhanced transduction of the target tissue at relatively modest vector doses^11^ and further improve the phenotypic outcome after gene therapy.^12^

An important focus of the AAV vector development field has been to fine‐tune the manufacturing process to augment the vector yield, purity, or its potency so that dose of vectors required per patient is low.^13^, ^14^ AAV transduction requires a reasonable multiplicity of infection of ~10^3^ to 10^5^ vector genomes (vg) per cell depending on cell type.^15^ However, for a clinical trial, an estimated 10^12^ to 10^14^ viral particles are essential to be efficacious during gene transfer.^15^, ^16^ This high vector dose requirement in the clinical settings has underscored the need for optimizing vector production and purification protocols. Thus, various strategies have been investigated.^2^, ^17^, ^18^ AAV vector production can be split in two stages: upstream cell culture and downstream purification and formulation. The conventional method relies on transfection of plasmid DNA into eukaryotic cells for clinical grade manufacturing of AAV.^19^ For clinical grade manufacturing of AAV, various producer or packaging cell lines,^16^ or a Baculovirus system using an insect cell line,^20^ or the Herpes Simplex Type I system are used.^21^ The conventional triple transfection protocol remains the most widely used protocol for clinical grade manufacturing of AAV vectors. The versatility of the system has been tested in a variety of AAV serotypes and transgene combinations. The method on an average can generate titers of about 10^11^ to 10^13^ vg/mL.^14^, ^22^ Significant efforts have been made to improvise the cell‐culture conditions and increase the vector yield. A modification to the transient transfection method, is the use of the stable rAAV producer cell lines for the large‐scale production of rAAV. Cell lines such as human embryonic kidney cells (HEK293) have been stably modified to express the E1 adenoviral helper gene, which circumvents the use of live adenovirus for helper function during AAV packaging.^14^, ^23^ Further, a stable cell line containing *rep* and *cap* genes has been established, that facilitates AAV production with mere adenoviral infection of the stable cell line, thus making large‐scale AAV production feasible.^24^

It is well known that AAV *rep* and *cap* genes play an important role during vector production.^23^, ^25^, ^26^ This necessitates that the *Rep* and *Cap* proteins be optimally expressed from the plasmid in order to achieve efficient vector replication and packaging.^26^ The *Rep* proteins are essential for genome rescue and its packaging, but the overexpression of these proteins are known to be cytotoxic.^25^, ^27^ Therefore, mutations at the first methionine codon [ATG (Met) to ACG (Thr)], which diminished the expression of *Rep* protein, yet enhanced the expression of capsid proteins to facilitate recombinant AAV assembly.^25^ A recent study has also employed hyperosmotic condition to improve the AAV vector yield by 1.8‐fold.^28^ Additionally, the purification protocols during AAV production has also seen significant advancements, with the inclusion of additional chromatography‐based purification methods,^29^ ultrafiltration of vectors,^30^ and polyethylene glycol‐based precipitation methods.^31^ While these protocols have largely relied on modifying extrinsic factors, the role of intrinsic cellular regulatory factors such as microRNA that can govern AAV packaging is not known.

MicroRNAs are known to play a major role in viral infectivity and pathogenicity.^32^, ^33^ Their effect on virus life cycle emanates from complex interactions involving host microRNAs^33^ or virally encoded microRNAs^34^ and their intracellular targets.^32^ miR‐122, a liver‐specific microRNA with its target in the 5′‐untranslated region (UTR) of hepatitis C virus (HCV) enhances the viral RNA translation^35^ and also protects HCV RNA genome from the exoribonucleases.^36^ Alternatively, certain atypical eukaryotic DNA to microRNA interactions, such as the role of miR‐373 inducing the gene expression from E‐cadherin promoter^37^ raises the possibility that promoter associated microRNAs could lead to increased gene expression from viral genes. In the context of AAV, one study has identified that cellular microRNAs such as hsa‐miR‐3687 are dysregulated at an early stage of wild type AAV2 infection in human cervical carcinoma (HeLa) cells.^38^ However, no data are available on the role of cellular small RNAs during recombinant AAV vector packaging.

Since our interest was to study the role of microRNA in context of vector packaging, we first wished to determine the identity of microRNAs that bind to the 145bp AAV inverted terminal repeat sequence (ITR) that flanks the genome. When compared to naturally occurring AAV, the recombinant AAV genome retains only the most essential viral element needed for successful replication or encapsidation, the ITRs, while the r*ep* and c*ap* genes are replaced by the therapeutic gene. The ITRs are also known to have both an enhancer and inhibitory roles in gene expression from AAV vectors. For example, AAV ITRs enable efficient and tissue‐specific expression of transgenes in *Xenopus* embryos.^39^ While on the contrary, ITR inhibits gene expression *via* its interaction with the cellular DNA damage‐sensing complex made up of Mre11, Rad50, and Nbs1 (MRN).^40^, ^41^ Since ITR is a known regulatory region for AAV gene expression, we utilized RegRNA,^42^ to identify potential microRNA target sites in AAV ITRs. Two microRNAs, hsa‐miR‐431 and hsa‐miR‐636, with a known regulatory function on cellular phosphokinases^43^, ^44^ were shortlisted from this analysis. These microRNAs were further incorporated in the AAV assembly protocol and the vectors tested for its therapeutic potential during AAV mediated suicide gene therapy.

## 2 MATERIALS AND METHODS

### 2.1 Cell lines

Human lymphoblastic myeloid leukemia cells (U937) cell line was a kind gift from Dr Vikram Mathews, Christian Medical College, Vellore, India. Human cervical carcinoma cells (HeLa) was purchased from American type culture collection (ATCC). AAV293 packaging cells were purchased from Stratagene and the murine lymphoblast (EL4) cells were obtained from the National Centre for Cell Science (NCCS) Pune, India. Cells were cultured in complete Iscove's modified Dulbecco's Medium (IMDM) (Gibco, Life Technologies) with 10% of fetal bovine serum (Gibco) at 37°C with 5% of CO_2_. This medium used for routine culturing was filter sterilized and supplemented with Ciprofloxacin (HiMedia Laboratories) and Piperacillin (MP Biomedicals) at 10 µg/mL each, before use.

### 2.2 MicroRNA prediction

To identify the microRNAs that may have steric and functional role during vector packaging, a RegRNA analysis was performed.^42^ A preliminary analysis predicted around 21 potential human microRNA binding sites within the ITR in AAV genome (Minimum Free Energy (MFE)> −16 and a *z* score >140). Of these, we chose two candidate microRNAs, miR‐431 and miR‐636, for their previously known biological function on cellular kinase targets^43^, ^44^ for further validation during vector packaging. The interaction of these microRNAs within the AAV ITR is shown in Figure S1.

### 2.3 Plasmid constructs

In order to overexpress each of the candidate microRNA selected, the pre‐microRNA sequence of miR‐431 or miR‐636 were retrieved from miRBase (http://www.mirbase.org/), amplified by PCR and inserted into pcDNA 3.1 plasmid (Addgene) downstream to the promoter by standard PCR/restriction enzyme‐based cloning methods. The insert sequence for the pre‐microRNAs is presented in Figure S2. The constructs thus generated were validated by their transfection in HeLa cells that revealed a 3‐ to 14‐fold increase in miR‐636 and miR‐431 levels, respectively (data not shown). An AAV plasmid containing either an inducible caspase 9 gene (iCasp9) or an enhanced green fluorescent protein (EGFP) as transgene^45^, ^46^ was utilized for packaging in the presence and absence of miR‐431 and miR‐636 constructs.

### 2.4 Packaging of AAV vectors by triple and quadruple transfection protocol

Recombinant AAV vectors were packaged in twenty 15 cm^2^ dishes containing the AAV293 cells at 70% confluency. Packaging was performed either by the conventional triple transfection protocol^47^ or by addition of the specific microRNA (quadruple transfection) with 0.1% of polyethylenimine (PEI, Polysciences, Inc) at 1:1 ratio of PEI: plasmid DNA. For the triple transfection protocol, plasmids pAAV2 or pAAV6 rep‐cap, pHelper, and gene of interest plasmid (p.ss.AAV‐CBa‐EGFP, p.sc.AAV‐CBa‐iCasp9) were each added at a concentration of 40 µg/plasmid/15 cm^2^ dish of the AAV293 packaging cells. In the quadruple transfection method, pcDNA‐miR431 or pcDNA‐miR636 were cotransfected with the triple plasmids at an equimolar concentration of 30 µg/plasmid/15 cm^2^ dish. After 72 hours, cells were harvested and lysed by repeated freeze‐thaw cycles, followed by incubation with benzonase (25 units/μL, Sigma Aldrich) and 4.8 M MgCl_2_ (Sigma) for 30 minutes at 37°C. Cell‐free virus was further purified by the iodixanol gradient ultracentrifugation and ion exchange chromatography. The purified viruses were concentrated using Amicon 10 kDa concentrator (Millipore) and stored in low retention tubes at −80°C. Packaged AAV vectors were quantified by a real‐time PCR‐based method described earlier.^48^ The physical particle titers of vectors were estimated from an average of six replicate samples and expressed as vector genomes (vg)/mL.

### 2.5 In vitro infectivity assay for reporter gene containing AAV serotype2 vectors

HeLa cells were seeded in a 24 well plate at a density of 3 × 10^4^ cells/well and incubated overnight at 37°C with 5% of CO_2_. AAV2 EGFP vectors packaged either by the triple or quadruple protocol were infected at a multiplicity of infection (MOI) of 5 × 10^3^ vgs/cell. Two days later, the transgene expression was assessed by flow cytometry in a BD Accuri C6 flow cytometer (BD Biosciences).

### 2.6 In vitro cytotoxicity assay for AAV6‐iCasp9 vectors packaged by either triple or quadruple protocol

To compare the cytotoxic potential of AAV6‐iCasp9 vectors packaged, U937 cells were either mock‐ (PBS) infected or infected with AAV6‐iCasp9 (triple) or AAV6‐iCasp9‐miR431 or AAV6‐iCasp9‐miR636 vectors, at an MOI of 5 × 10^4^ vgs/cell. A day later, cells were treated with a 10 nmol/L concentration of a dimerizer drug AP20187 (ARIAD Pharmaceuticals).^49^ Cell viability was assessed using Cell Titer‐Glo Luminescent cell viability assay kit as per manufacturer's instructions (Promega Corporation).^45^

### 2.7 Transcriptome Sequencing

To assess the modulation of cellular transcriptome during vector packaging, AAV293 cells in 15 cm^2^ dishes (n = 4, per condition) were transfected by either triple or quadruple transfection methods, with pAAV2 rep‐cap, pHelper, AAV‐EGFP plasmids, and with either pcDNA miR‐431 or pcDNA miR‐636 plasmids in equimolar concentration as described above. Twenty‐four hours later, cells were harvested and the total RNA was isolated using TRIzol Reagent (Thermo Fisher) as per manufacturer's protocol. RNA samples from conventional triple transfection protocol to generate AAV2‐EGFP wild type was designated as Control or C, while packaging cells with miR‐431 or miR‐636 was labeled as treated condition 1 (T1) and treated condition 2 (T2), respectively. RNA samples in duplicates from two sets of packaging was further analyzed by a transcriptome profiling on an Illumina Hiseq 2500 platform (Bionivid Technologies). The raw data obtained from this analysis are submitted in SRA archive (https://submit.ncbi.nlm.nih.gov/subs/sra/SUB6272613) (Table S1).

### 2.8 Differential gene expression analysis

RNA isolated from each of the conditions was processed to generate RNA libraries using Illumina HighSeq 2500 (Illumina). A paired‐end transcriptome sequencing was performed, to generate 30‐40 million reads for each sample with a read length of 100 bp.^50^ Raw reads obtained were filtered to exclude low complexity reads and those containing adaptor sequences. These reads were then aligned against known transcripts of Hg38 using TopHat‐2.0. The high‐quality reads were then filtered and used for differential gene expression of T1 (+miR‐431) and T2 (+miR‐636) conditions in comparison to the control samples (triple transfection). The differentially expressed genes (DEGs) were then analyzed by Gene Ontology (GO)^51^ and Kyoto Encyclopedia of Genes and Genomes (KEGG)^52^ enrichment analysis. Finally, genes with a *P* ≤ .05 and fold change ≥2 were considered as significantly different between the test (T1 and T2) and control condition.

### 2.9 Western blot analysis

AAV293 cells were transfected by triple and quadruple transfection methods with AAV2‐EGFP vectors and miR‐431 or miR‐636, as detailed above. Cells were washed twice, lysed in radioimmunoprecipitation assay (RIPA) buffer supplemented with protease inhibitor (Complete protease inhibitor cocktail tablets, Roche) for 30 minutes at 4°C with intermittent agitation, followed by sonication. The protein concentration was measured by the BCA protein assay kit (Pierce, Thermo Fisher). About 30 μg of total cell lysate was loaded in each well and further resolved by 10% of SDS‐PAGE analysis. After a transfer, the membrane was probed with a primary antibody to mitogen activated protein kinase (MAPK) (1:1000 dilution, p44/42 MAPK (Erk1/2), Cell Signaling Technologies). β‐actin was used a loading control (1:5000, Abcam). The immune‐reactive bands were then visualized by a chemiluminescent method (Pierce ECL assay, Thermo Fisher).

### 2.10 Animal procedures

C57BL/6J mice were purchased from Jackson Laboratory. Animals were maintained in accordance with the guidelines of CPCSEA (Committee for the Purpose of Control and Supervision of Experiments on Animals), with free access to food and water.

### 2.11 Evaluation of suicide gene transfer vectors packaged by triple or quadruple method in a syngeneic model of lymphoma

A syngeneic model of lymphoma was generated by intramuscular administration of EL4 cells in C57BL/6J mice as described earlier.^53^ Approximately, 2 × 10^6^ EL4 cells were administered into each mice in a 100 μL volume twice , on day 0 and day 7. Ten days later, palpable tumors were observed and the tumor volume was measured with Vernier calipers as described earlier.^54^ Subsequently, tumor bearing mice were randomized to receive: PBS (mock‐administered, n = 5) or suicide gene containing AAV vectors (AAV6‐iCasp9, AAV6‐iCasp9‐miR431, or AAV6‐iCasp9‐miR636) at a dose of 2 × 10^10^ vgs per animal (n = 5 per group), intratumorally. The vector‐administered animals received three consecutive doses of AP20187 at 1 mg/kg body weight, intraperitoneally. The experiment was terminated when the tumors reached 25‐28 mm in diameter, or if the mice had succumbed to the tumor. At the end of the experiment, animals were euthanized humanely, the tumor volumes were recorded and the tumor samples harvested as previously described.^54^, ^55^ The tumor tissue was further processed for histological analysis. A detailed experimental outline is depicted in Figure S3.

### 2.12 Histological analysis

Tumors from the AAV6 vector‐administered (AAV6‐iCasp9, AAV6‐iCasp9‐miR431, and AAV6‐iCasp9‐miR636) or control group were harvested, washed briefly with PBS, and fixed in 10% of buffered formalin. Samples were further processed by paraffin embedding and tissue sectioning (8 μm). Tumor sections (n = 2 per animal; 3 animals per group) were stained with hematoxylin and eosin. An unbiased examination for apoptosis was performed by a certified pathologist, as previously described.^56^

### 2.13 In situ TUNEL assay

Tumor samples excised from mice were washed briefly with PBS and fixed in 10% of buffered formalin. Samples were then cryopreserved by mounting in an OCT medium (Sigma Aldrich). About, 5 µm sections were cut with a cryotome (Leica). Sections were rinsed in PBS and fixed in 4% of paraformaldehyde for 1 hour at 37°C. An in situ TUNEL staining to detect apoptotic cells and a counter staining with 4′, 6‐diamidino‐2‐phenylindole (DAPI, Thermo Fisher) was performed according to the manufacturer's protocol (Roche). Images were captured in a Leica DM5000B microscope.

### 2.14 Statistical analysis

Data are presented as mean ± SD. Multiple comparisons between the test and control groups was performed by analysis of variance (ANOVA) tests using GraphPad prism v7.0 (GraphPad Software). A *P* < .05 was considered to be statistically significant.

## 3 RESULTS

### 3.1 Vector yield

The primary objective of this study was to investigate the potency of recombinant AAV vectors generated by both the triple and quadruple transfection protocols. However, we also compared the yield of AAV vectors generated in both these conditions. All the vectors were packaged under similar culture conditions with an equimolar concentration of plasmid DNA and with the same lot of AAV293 packaging cells. A total of six vectors, including AAV6‐iCasp9, AAV6‐iCasp9‐miR431, and AAV6‐iCasp9‐miR636 or AAV2‐EGFP, AAV2‐EGFP‐miR431, and AAV2‐EGFP‐miR636 were generated and further quantified by a real‐time PCR method. An AAV2 reference material (RSM), ATCC VR‐1616 was used as an internal control. Our data (Table 1) show that the vectors prepared by quadruple transfection protocol had titers similar to those generated by the triple transfection protocol. This further suggests that the overexpression of microRNA during packaging in AAV293 cells, does not compromise the packaging ability of AAV vectors.

**Table 1:** Quantification of vector yield when packaged by quadruple and triple transfection protocols

Vector	Titer (vgs/mL)
AAV6‐iCasp9	3.67 ± 0.56 × 10^11^
AAV6‐iCasp9‐miR431	6.27 ± 1.18 × 10^11^
AAV6‐iCasp9‐miR636	6.46 ± 0.5 × 10^11^
AAV2‐EGFP	4.14 ± 0.21 × 10^11^
AAV2‐EGFP‐miR‐431	1.49 ± 0.57 × 10^11^
AAV2‐EGFP‐miR‐636	1.51 ± 0.35 × 10^11^

Note

The data are a mean of two independent titrations by quantitative PCR.

### 3.2 Transduction efficiency of vectors in vitro

**Figure 1:**
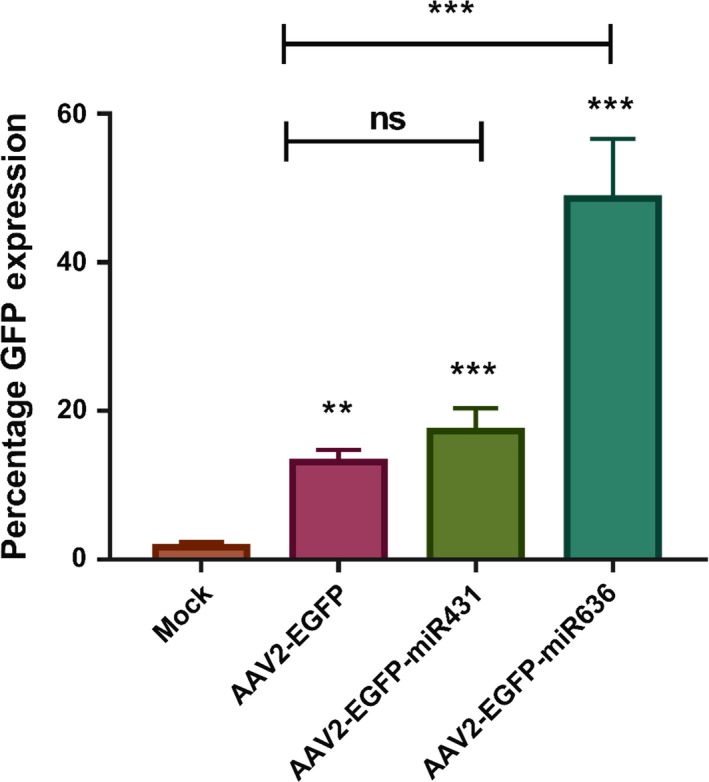
Transduction efficiency of AAV vectors packaged by the triple and quadruple transfection protocols: HeLa cells were infected with AAV2‐EGFP vectors that were packaged in the presence or absence of specific microRNAs. Flow cytometry was performed 2 d later. The data obtained are expressed as mean ± SD (n = 6). **P < .01; ***P < .001; ns‐not significant. The data set is cumulative of two biological replicates and three technical replicates for each experiment

To evaluate the phenotypic potential of AAV vectors packaged in the presence of microRNAs, ~5 × 10^3^ vgs of AAV vectors were used to infect HeLa cells. Two days later, the GFP expression was measured by flow cytometry. Our data show that vectors packaged in presence of microRNAs, had a significant increase in transduction. The maximal increase was noted with vectors packaged in the presence of miR‐636, with a 3.69‐fold higher transduction noted in HeLa cells in comparison to cells infected with AAV2‐EGFP vectors generated by the conventional triple transfection method (Figure 1). These data highlight that vectors packaged by a quadruple transfection method and in the presence of miR‐636, have a superior transduction efficiency.

### 3.3 In vitro cytotoxicity assay in leukemic cells

**Figure 2:**
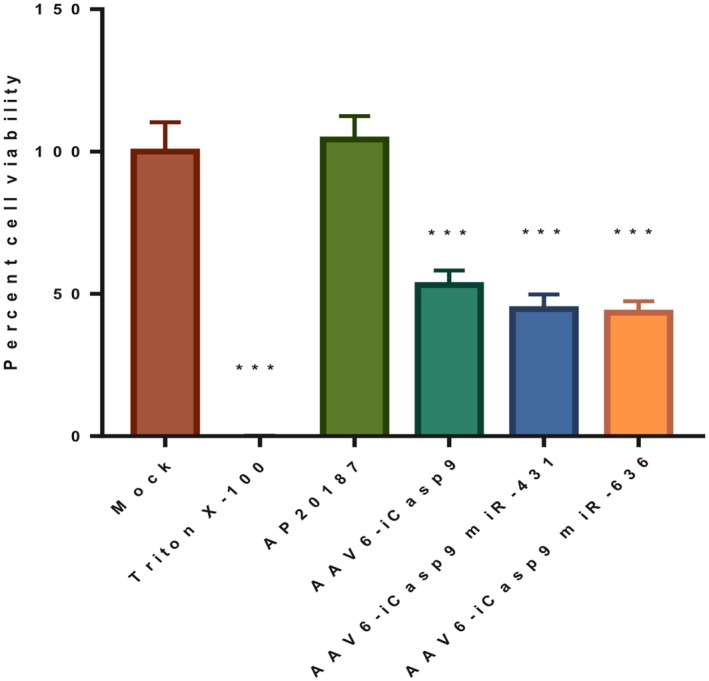
In vitro cytotoxicity of iCasp9 containing AAV vectors packaged with triple and quadruple transfection conditions: U937 cells were infected with vectors containing iCasp9 gene/AP20187 (10 nmol/L). Two days later an ATP dependent luminescence assay was performed to assess the viability of U937 cells. Representative data set from three biological replicate experiments with four technical replicates is shown. Triton X‐100: positive control; AP20187, drug control. ***P < .001 vs mock‐treated cells

To examine whether a therapeutic gene (iCasp9 suicide gene) containing AAV packaged in presence of microRNA is effective, we evaluated its cytotoxicity in U937 cells. Cells were infected with either AAV6‐iCasp9‐WT vectors prepared by triple transfection or AAV6‐iCasp9‐miR431/AAV6‐iCasp9‐miR636 vectors prepared by a quadruple transfection at an MOI of 5 × 10^4^ vgs per cell. After treatment of cells with a dimerizer drug, both AAV6‐iCasp9‐miR431 and AAV6‐iCasp9‐miR636 vector infected U937 cells had a similar cytotoxic profile (~57%), whereas the cytotoxicity was lower (~47%) in cells infected with AAV6‐iCasp9‐WT vectors (Figure 2). This suggests that AAV6 vectors packaged in the presence of microRNA demonstrate a modest increase in cytotoxicity against the leukemic cells. Our data also reflect a possible saturation effect in cytotoxicity with a higher dose of vectors (5 × 10^4^ vgs/cell) employed for this assay.

### 3.4 Transcriptome analysis reveals differentially expressed genes when AAV vectors are packaged in presence of microRNA

**Figure 3:**
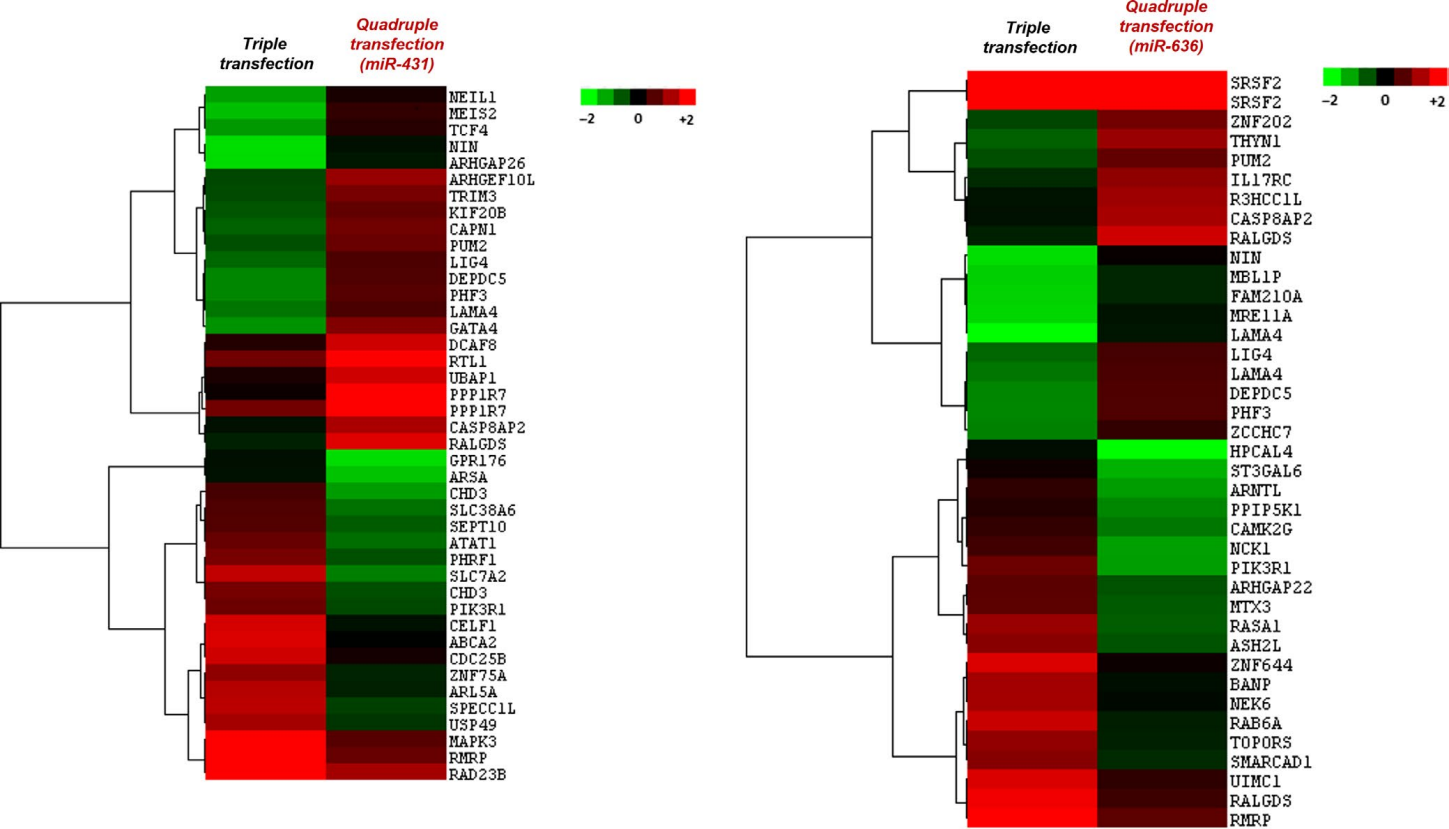
Differentially expressed genes between triple and quadruple transfection protocols: A transcriptomic analysis was performed on total RNA isolated from packaging cells transfected with packaging plasmids for generating AAV2‐EGFP vectors either by a triple or quadruple transfection. The hierarchical clustering analysis depicts the heat map of dysregulated genes and their identities for each of the conditions tested. The data generated are from a mean of two independent biological replicate samples

Considering the incorporation of regulatory microRNAs in the quadruple packaging protocol, it was crucial to delineate the alterations to host cellular gene expression during AAV packaging. Our transcriptome analysis revealed that in comparison to the conventional triple transfection protocol (AAV2‐EGFP; control), 42 transcripts were dysregulated in AAV293 cells when vectors were packaged in the presence of miR‐431, while 39 genes were dysregulated when miR‐636 was included in the packaging protocol (Figure 3).

**Figure 4:**
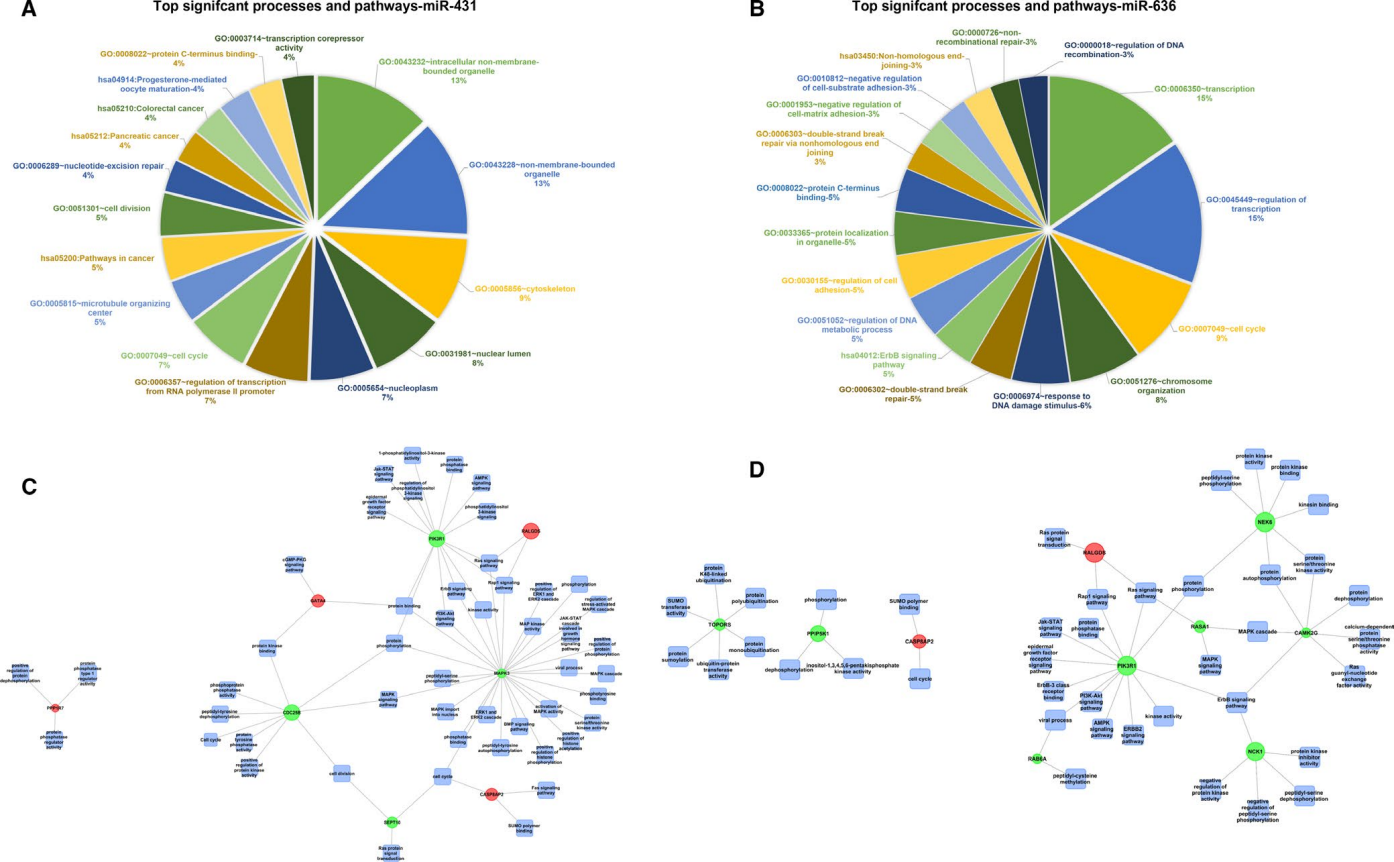
Functional annotation and network analysis: The pie charts represent the major functional annotations reflecting significant processes and pathways for the dysregulated genes in A, miR‐431 incorporated quadruple transfection method vs control (triple transfection) and B, miR‐636 incorporated quadruple transfection method vs control. C,D Network analysis shows that multiple genes associated with MAPK signaling pathway are significantly downregulated in the quadruple transfection method either in the presence of miR‐431 (C) or miR‐636 (D), respectively

To further compare the dysregulated gene sets from both the conditions, we performed a functional annotation using DAVID NIH data resource for Gene Annotation Biological Process, Gene Annotation Cellular Component, Gene Annotation Molecular Function and human genome referred KEGG Pathway (Figure 4A,B). Retro transposon‐like protein 1 (RTL1) and serine/arginine‐rich splicing factor 2 (SRSF2) genes, which flanked the pre‐microRNA constructs of miR‐431 and miR‐636, respectively, were found to be upregulated by 6.23‐ and 2.7‐fold, respectively, in conditions where these microRNA were cotransfected. A more detailed analysis further revealed that a large fraction of genes dysregulated when miR‐431 (64%, 27/42) or miR‐636 (61%, 24/39) were included, belonged to the family of phosphoproteins (Table 2). Furthermore, a number of dysregulated genes mapped to various cellular signaling pathways (Figure 4C)*.* For example, CDC25B (Cell Division Cycle 25B, ‐2.19‐fold) and MAPK3 (Mitogen‐Activated Protein Kinase 3, ‐2.2‐fold) genes were downregulated in miR‐431 treated condition while in miR‐636 treated condition, the CAMK2G (Calcium/Calmodulin Dependent Protein Kinase II Gamma, ‐2‐fold) and RASA1 (RAS P21 Protein Activator **1**, ‐2.9‐fold) genes were downregulated. All of these genes are involved in the MAPK (Mitogen‐activated protein kinase) signaling pathway (Figure 4D). Similarly, PIK3R1; a member of the JAK‐STAT pathway was observed to be downregulated by 2.1‐fold and 3.1‐fold in miR‐431 and miR‐636 treated conditions, respectively. Other signaling pathways genes that were identified include like PI3K‐AKT signaling pathways. It is well known that these signaling pathways (MAPK, PI3k‐AKT or JAK‐STAT pathway) regulate their signal transduction via phosphorylation.^57^ The involvement of these pathways in the quadruple packaging protocol indicates alterations to the phosphorylation status of the packaging cells. The specific downregulation of nodal genes within these pathways also suggests that vectors that are packaged in these conditions could have reduced phosphorylation when compared to vectors generated by the triple transfection protocol. Other than phosphorylation, genes involved in prominent posttranslational modification (PTM) like SUMOylation (USP49, Ubiquitin carboxyl‐terminal hydrolase 49, ‐2.5‐fold) and ubiquitination (UBAP1, ubiquitin associated protein 1, 2.1‐fold; UIMC1, ubiquitin interaction motif containing 1, ‐2.02‐fold) were also dysregulated in the quadruple transfection protocol. Our gene expression analysis thus indicates that the phosphorylation status of the packaging cells and its possible impact on PTMs in viral capsid could be a major determinant for the increased transduction seen with vectors generated by quadruple transfection. However, a specific analysis of the phosphoproteome of the vector capsids generated by quadruple transfection protocol will be necessary to confirm this phenomenon.

**Table 2:** Functional enrichment analysis

Major keywords	Count	Fold enrichment	*P* value
(A)			
Sequence variant	29	1.284	2.28E‐02
Phosphoprotein	**27**	**1.986**	**1.22E‐05**
Alternative splicing	26	1.855	9.77E‐05
Splice variant	26	1.851	1.02E‐04
Cytoplasm	12	1.924	3.00E‐02
Nucleotide‐binding	8	2.535	2.99E‐02
Cell cycle	6	6.954	1.39E‐03
Cytoskeleton	5	4.201	2.75E‐02
Cell division	4	8.096	1.21E‐02
IPR019787:Zinc finger, PHD‐finger	3	16.799	1.30E‐02
IPR001965:Zinc finger, PHD‐type	3	15.866	1.45E‐02
SM00249:PHD	3	10.808	2.90E‐02
(B)			
Alternative splicing	25	2.072	5.22E‐06
Splice variant	25	2.067	5.48E‐06
Sequence variant	25	1.285	3.45E‐02
Phosphoprotein	**24**	**2.050**	**1.64E‐05**
Polymorphism	24	1.289	4.39E‐02
Nucleus	15	2.173	2.67E‐03
Transcription regulation	8	2.450	3.31E‐02
Transcription	8	2.397	3.67E‐02
Nucleotide‐binding	7	2.576	4.30E‐02
Ubl conjugation	5	5.276	1.26E‐02
Cell cycle	4	5.384	3.44E‐02
SH2 domain	3	16.770	1.29E‐02
IPR000980:SH2 motif	3	15.008	1.59E‐02
SM00252:SH2	3	10.669	2.92E‐02
DNA repair	3	9.746	3.56E‐02
DNA damage	3	9.080	4.04E‐02
Sh3 domain	3	8.864	4.22E‐02
Chromatin regulator	3	8.739	4.33E‐02
Domain:SH2 2	2	123.310	1.56E‐02
Domain:SH2 1	2	123.310	1.56E‐02

Note

The dysregulated elements in both the conditions were classified based on the keywords obtained from DAVID NIH tool. The table describes the keywords, the gene numbers that cluster to the keyword, and their relative fold enrichment for (A) cells packaged with quadruple method with the addition of miR‐431 vs control (triple transfection) and (B) with the addition of miR‐636 versus control.

Bold font indicates the phosphoproteins, that are further subject of investigation of the study.

### 3.5 Immunoblotting confirms alteration to MAPK pathway during vector generation

**Figure 5:**
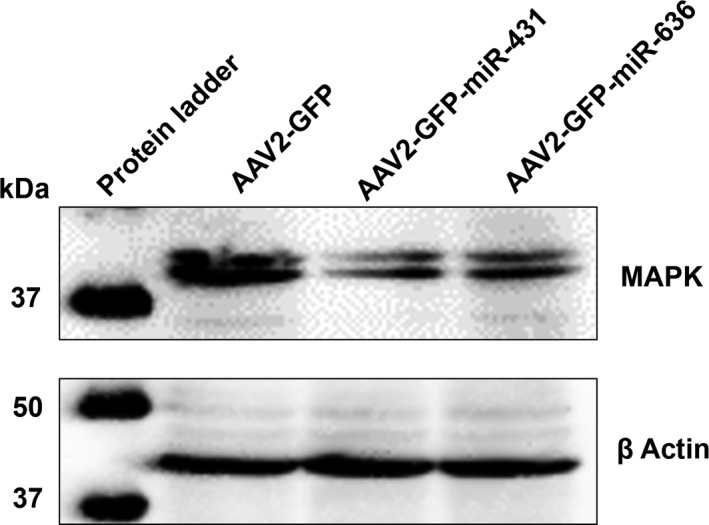
Western blotting to detect MAPK activity: AAV293 packaging cells were collected 24 h after a triple or quadruple transfection (+miR‐431 or miR‐636) for generation of AAV2‐EGFP vectors. The protein lysates were resolved by SDS‐PAGE analysis and probed for p44/42 MAPK proteins. β‐actin was used as a loading control. Data are representative of three independent experiments

To further validate the involvement of MAPK pathway during AAV packaging, we performed an immunoblotting assay with a site‐specific antibody [p44/42 MAPK (Erk1/2)]. This antibody specifically recognizes phosphorylated (Thr202/Tyr204) Erk1/2, indicating the activation of the MAPK signaling pathway, as previously reported.^58^ Our data presented in Figure 5, demonstrate that phosphorylated forms of MAPK are downregulated in the quadruple packaging method.

### 3.6 Therapeutic validation of AAV6‐iCasp9 vectors packaged by quadruple transfection in a murine lymphoma model

**Figure 6:**
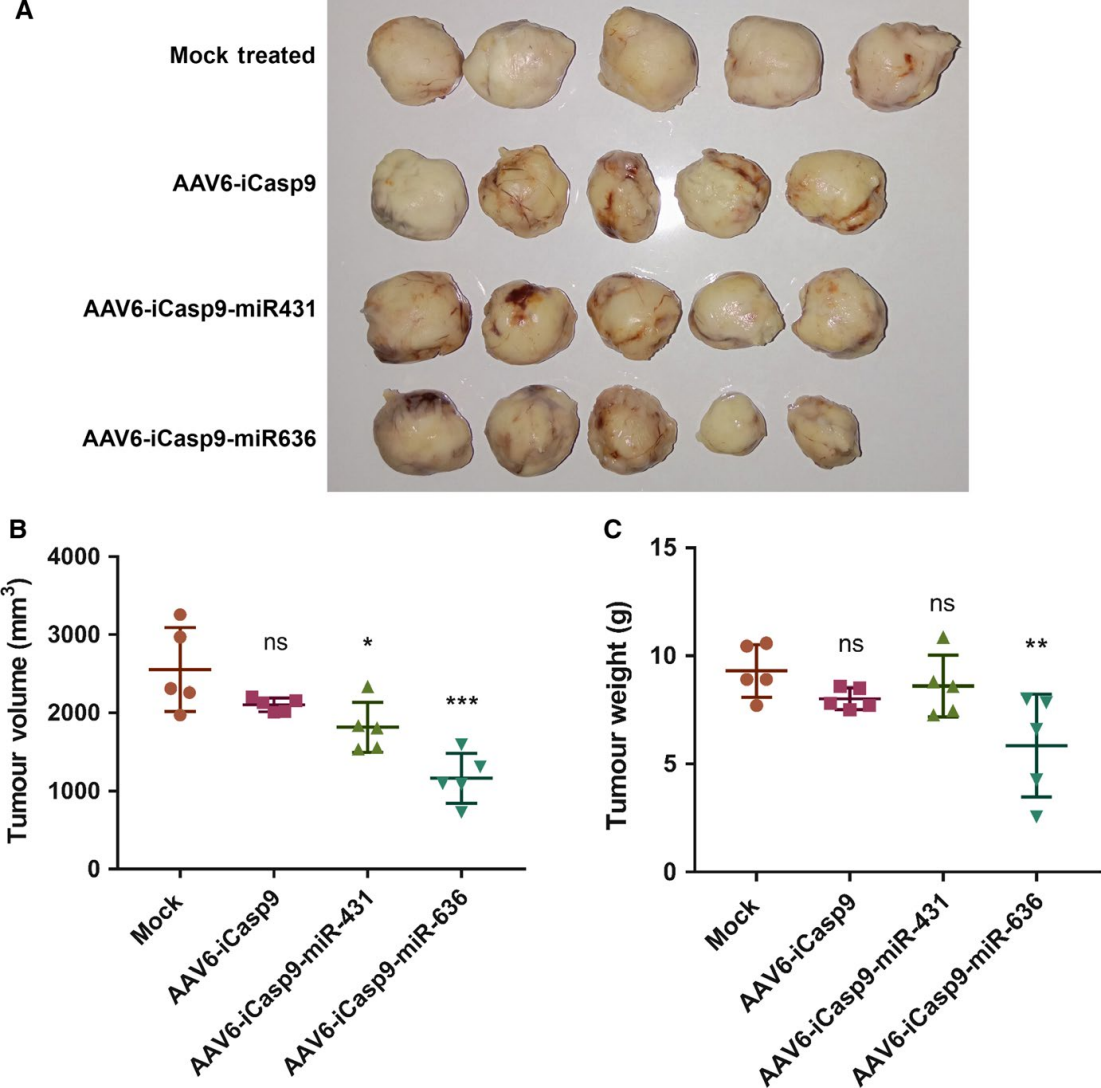
Phenotypic effect of AAV6‐iCasp9 suicide gene therapy in a murine model of lymphoma: C57BL6/J mice were administered with 2 × 10^6^ EL4 cells by intramuscular injections. Groups of mice (n = 5) were treated intratumorally with AAV6‐iCasp9 vectors generated by either the triple or quadruple transfection (+miR‐431 or miR‐636) protocols. A, Image of harvested tumors from all the treatment groups demonstrate a significant regression of tumor with AAV6‐iCasp9 vectors generated in the presence of miR‐636. B, Tumor volume and C, tumor weight was measured on day 8 after suicide gene therapy. The mean value (±SD) of these parameters plotted from five mice per group are shown. ns‐not significant, *P < .05, **P < .01, ***P < .001 vs mock‐treated group

To further validate the therapeutic efficacy of vectors packaged with quadruple transfection protocol, we performed an intratumoral delivery of the AAV6‐iCasp9 vectors (2 × 10^10^ vgs per animal) in a murine lymphoma model. EL4 is an aggressive tumor cell line capable of producing visible tumors in the mice within 5‐9 days of cell administration.^53^ In our study, all the animals (n = 5) in the control (mock‐injected) group succumbed by day 18 after EL4 cell challenge, however, the survival in the vector treated mice (n = 5 animals/group; total = 15 animals) was about 60%‐80% at the same time point. A gross examination of tumor revealed a visible regression in tumor volume of the animals that received a combination of AAV6‐iCasp9‐miR636 vectors and the dimerizer drug, AP20187, in comparison to the mock‐administered animals (Figure 6). Furthermore, mice that received AAV6‐iCasp9 vectors packaged in the presence of miR‐636 had a considerable reduction in tumor weight (~1.5‐fold vs mock, *P* < .01) and tumor volume (~2.2‐fold vs mock, *P* < .001) (Figure 6A‐C)*.* In contrast, administration of either AAV6‐iCasp9 or AAV6‐iCasp9‐miR431 vectors had only a modest effect on the tumor volume in comparison to untreated mice (Figure 6A‐C).

**Figure 7:**
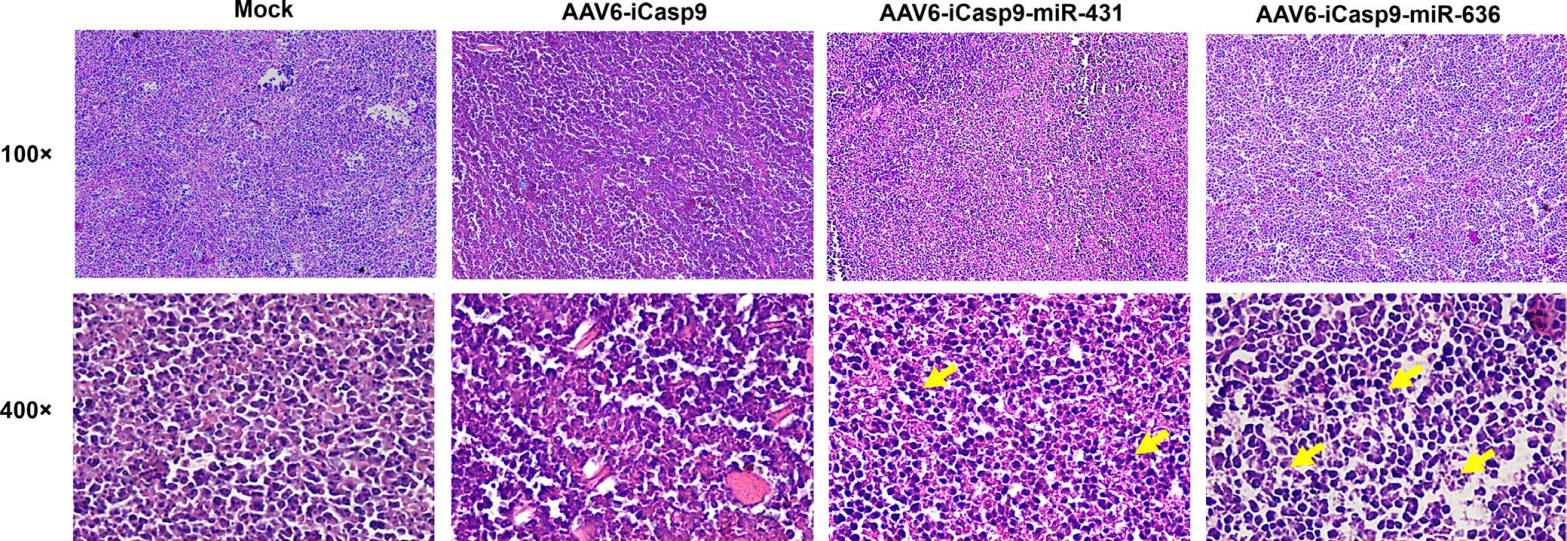
Histopathological analysis of murine lymphoma tissue treated with AAV6‐iCasp9 vectors: Hematoxylin and eosin staining was performed on tumor tissues harvested from mock‐injected and AAV6‐iCasp9‐administered mice. When observed microscopically, tumor tissue revealed a notable pyknosis and apoptosis in tumors harvested from animals received AAV6‐iCasp9 vectors followed by AP20187 administration (denoted by arrows in representative images). Magnification‐100X and 400X

Furthermore, we performed a morphological characterization of the harvested tumors by hematoxylin and eosin staining. In case of tissue sections from the mock‐treated group, only a few apoptotic cells were seen (Figure 7). A higher proportion of apoptotic cells (marked by arrow) and tissue disintegration was observed across the tumors that had been injected with AAV6‐iCasp9‐miR636 vectors followed by AP20187 treatment. These data further confirmed the superior suicide gene delivery seen with AAV6 vectors packaged by the quadruple transfection in the presence of miR‐636.

**Figure 8:**
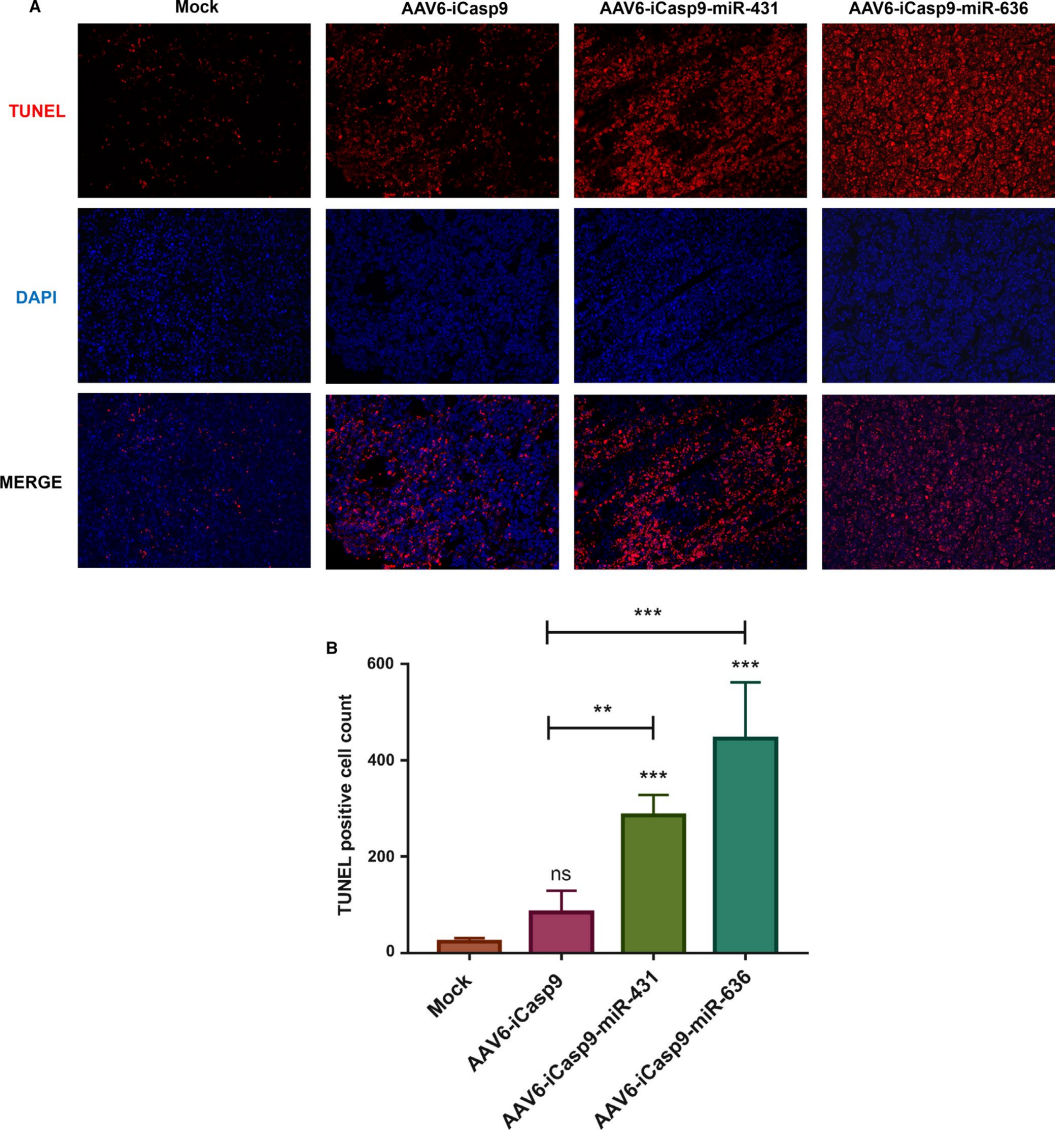
TUNEL assay to detect apoptotic activity in murine lymphoma sections after AAV6‐iCasp9 suicide gene therapy: A, Representative Images of TUNEL stained tumor sections from murine lymphoma sections after suicide gene therapy is shown. Nuclei stained in red color indicates the TUNEL positive apoptotic cells. Cell nucleus was counter stained by DAPI. B, The number of apoptotic cells were higher in tumors harvested from animals that received vectors generated by the quadruple transfection method. ns, not significant, **P < .01, ***P < .001 vs mock‐treated group

To further validate these findings, we performed an in situ TUNEL assay which detects DNA fragmentation in apoptotic cells. TUNEL positive cells is demonstrated by a red staining of the tumor cell nuclei. As can be seen in Figure 8, a substantial increase in apoptotic cells can be noted in tumor sections of mice administered with AAV6‐iCasp9 vectors generated in the presence of miR‐636 (18.9‐fold vs mock group, *P* < .001). This apoptotic activity in tumor sections was relatively higher than in tumors harvested from animals treated with AAV6‐iCasp9‐miR431 (12.2‐fold) or AAV6‐iCasp9 vectors (1.6‐fold)*.*

## 4 DISCUSSION

A critical component in augmenting the potential and reach of AAV‐based gene therapy is to carefully design and develop protocols that can ensure better outcomes with the recombinant vectors generated.^59^ The potency of the vector is the intrinsic and the most determining factor that affects its overall therapeutic efficacy.^19^ We report here a modified vector production method that has incorporated a fourth plasmid containing a microRNA to the conventional triple transfection method. This microRNA‐based AAV assembly and packaging method, has shown a significant improvement in transduction efficiency and the phenotypic outcome of AAV mediated suicide gene therapy. This protocol is advantageous as it is based on the existing triple transfection method with only the addition of a fourth plasmid (eg miR‐636). Thus, this method can be easily co‐opted with the current vector generation methods.

We have also provided a proof of concept for the potential of regulatory microRNAs in improving vector infectivity. In our limited global transcriptome profiling, 24 hours after transfection, we identified that cellular phosphoproteins play a predominant role during AAV assembly. Based on this finding and the fact that miR‐636 and miR‐431 is predicted to bind to AAV ITRs, we incorporated these microRNAs in the packaging process. MicroRNAs have been previously employed to enhance the antitumor activity in preclinical models.^60^ Interestingly, hsa‐miR‐431 has been studied in association with colorectal cancer (CRC). This microRNA has been shown to influence the JAK‐STAT signaling in CRC and further suppress the PI3K‐Akt and MAPK pathways to inhibit cell viability.^61^ Hsa‐miR‐636 is also known to act as a tumor suppressor in human hepatocellular carcinoma by targeting *ANT2* and *Ras* genes thereby inactivating the Ras signaling pathway.^44^ While these data are suggestive, a comprehensive transcriptomic and global microRNA analysis in the vector packaging cells from the earliest time point after a plasmid transfection, up to cell lysis (1‐72 hours after transfection) is needed to understand their impact on vector production. Such data are also likely to pinpoint specific regulatory microRNAs that can improve vector assembly, yield or its infectivity.

Our finding that cellular phosphoproteins such as those involved in MAPK signaling can influence vector production is supported by previous studies. A method to improve the infectivity of AAV vectors, by either cotransfection of a protein phosphatase 5 (PP5) gene containing plasmid during vector generation^62^ or when used as an adjunct helper PP5 virus,^63^ substantially lowered the dose of factor IX vector required to achieve a phenotypic rescue in vivo. Thus, the concept of blocking phosphorylation of specific cellular proteins through either a phosphatase enzyme^62^ or through specific microRNA as described here, appears to be beneficial in enhancing the infectivity and outcome of AAV mediated gene therapy.

Recently, data on the diversity of PTMs in recombinant AAV1‐rh10 serotypes during its production have been reported.^46^ In a limited validation with AAV2 serotype, PTMs such as glycosylation and SUMOylation^46^ at specific sites were demonstrated to be rate‐limiting for both transduction and generation of AAV vectors. Similarly, the impact of other PTMs such as ubiquitination on viral infection, has been studied widely, in multiple AAV serotypes.^64^, ^65^ We thus speculate that addition of microRNA during recombinant AAV production can modulate some of these PTMs and further improve its infectivity. Our study also has the following limitations. The utility of the microRNA‐based vector production protocol needs to be studied comprehensively, by employing multiple transgenes and various AAV serotypes. Furthermore, an advanced mass spectrometry‐based characterization of phosphoprotein content of AAV capsids^46^ generated by quadruple transfection protocol is likely to shed light on the mechanistic basis of this enhanced infectivity seen during suicide gene transfer.

## 5 CONCLUSIONS

We have developed a simple strategy to improve the transduction potential of AAV vectors by incorporating miR‐636, during vector assembly. This method improved the therapeutic outcome during suicide gene delivery in a syngeneic model of murine lymphoma. This strategy, needs to be comprehensively validated further in multiple disease models in vitro and in vivo*.*

## 6 ETHICS STATEMENT

The animal studies were performed after approval by Institute Animal Ethics Committee at Indian Institute of Technology, Kanpur, India.

## CONFLICT OF INTEREST

The authors state that “No competing financial interests exist.”

## AUTHOR CONTRIBUTIONS

NK, SC, HS, SB performed the experiments; GRJ designed the study; NK and GRJ wrote the manuscript.

## Data Availability

The data that support the findings of this study are available on request from the corresponding author.
