# Supplementary material for: MicroRNA‐based recombinant AAV vector assembly improves efficiency of suicide gene transfer in a murine model of lymphoma
Source: Cancer Med. 2020 Feb 28;9(9):3188–201. doi: 10.1002/cam4.2935 (PMC7196056; doi:10.1002/cam4.2935)
Supplement: Supplementary file 1 [file CAM4-9-3188-s001.docx]

***Supporting Information***


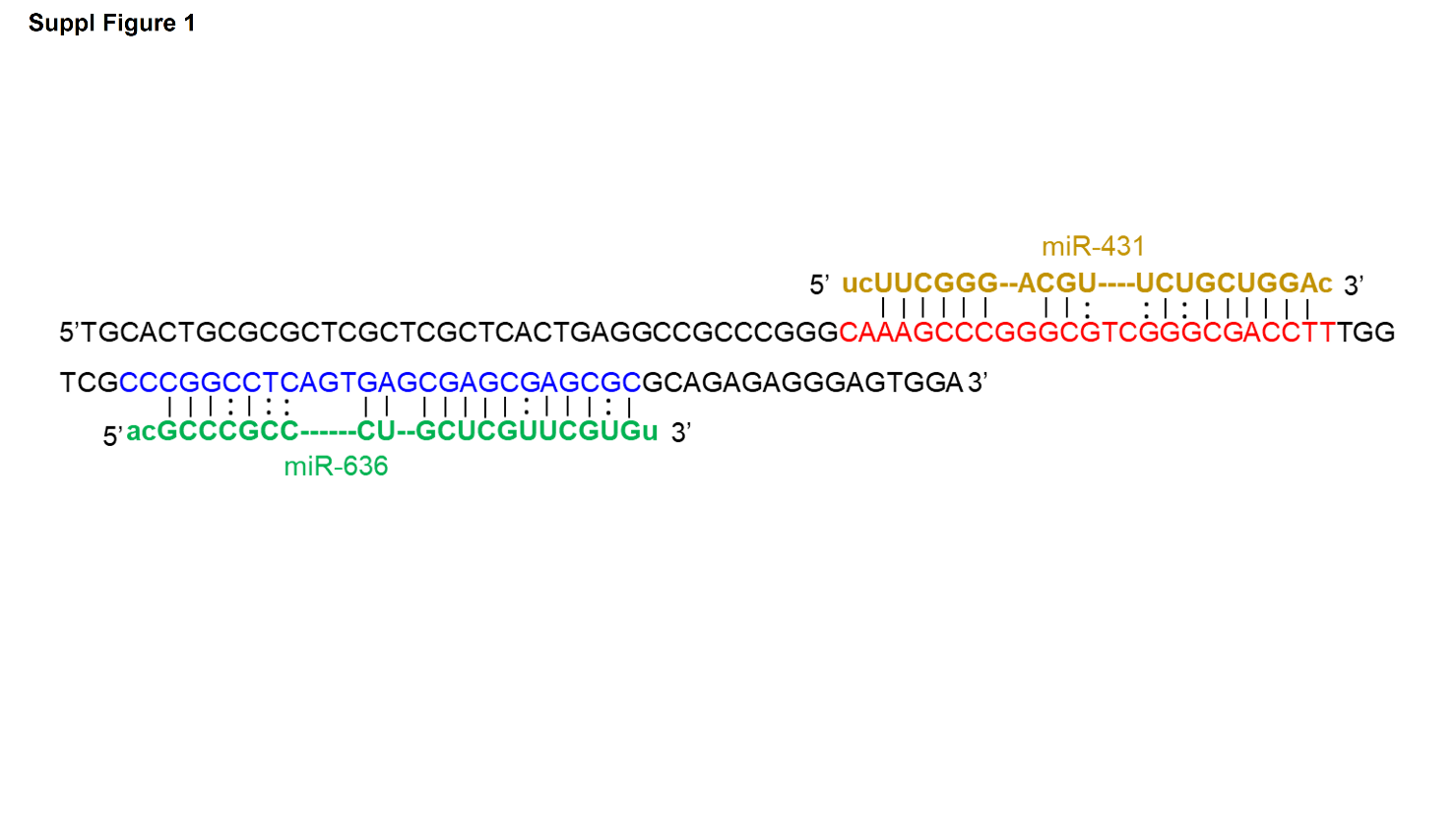


***Figure S1: Interaction of hsa-miR-431 and hsa-miR-636 with AAV2 inverted terminal repeat region (ITR).***

***
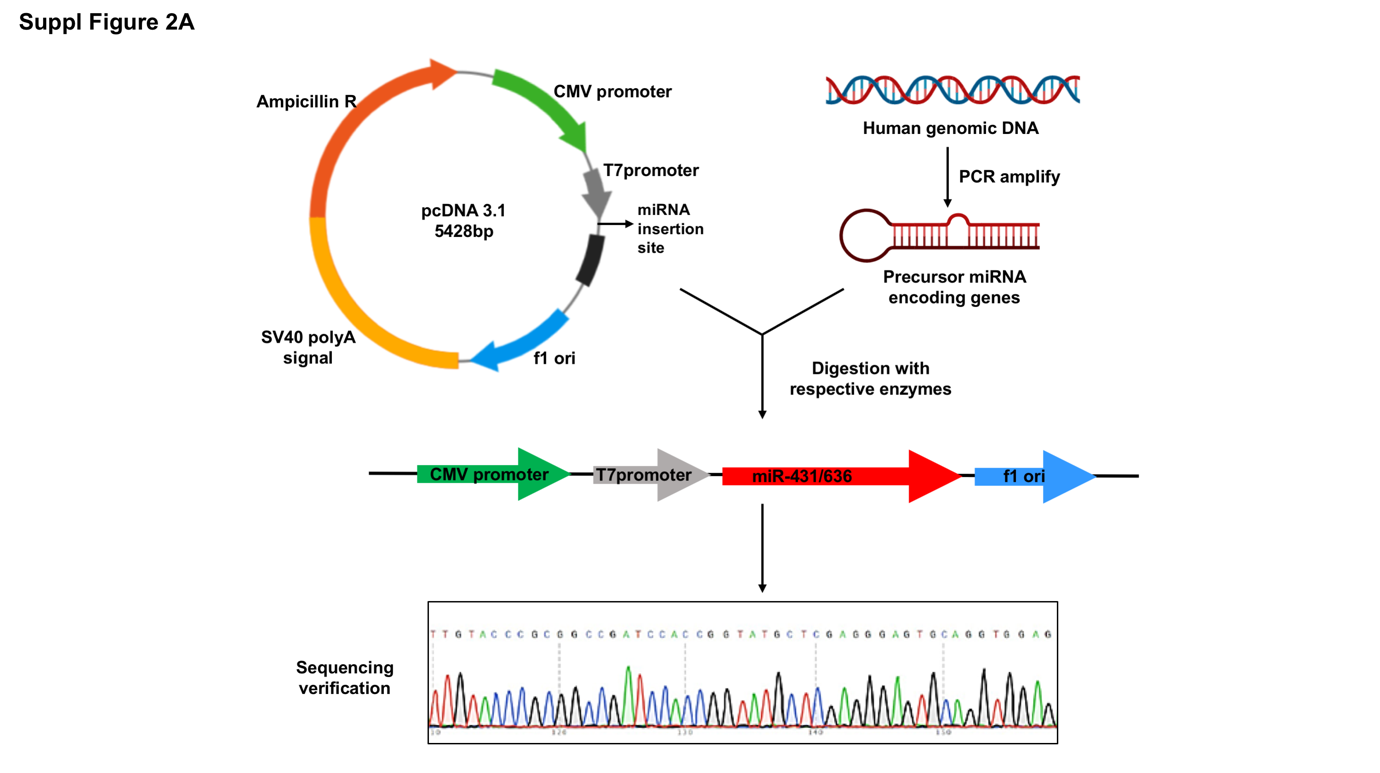
***

***
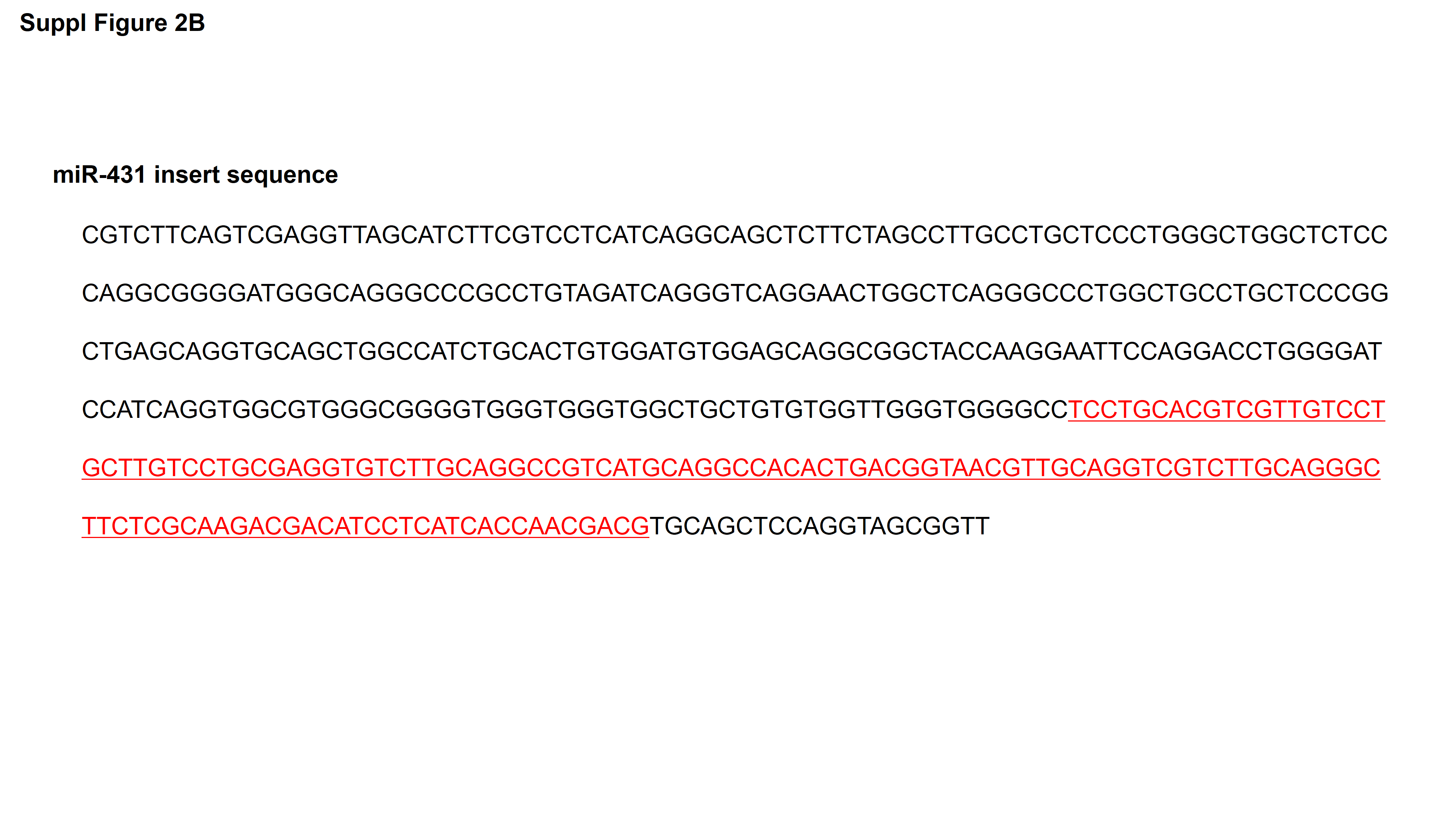
***

***
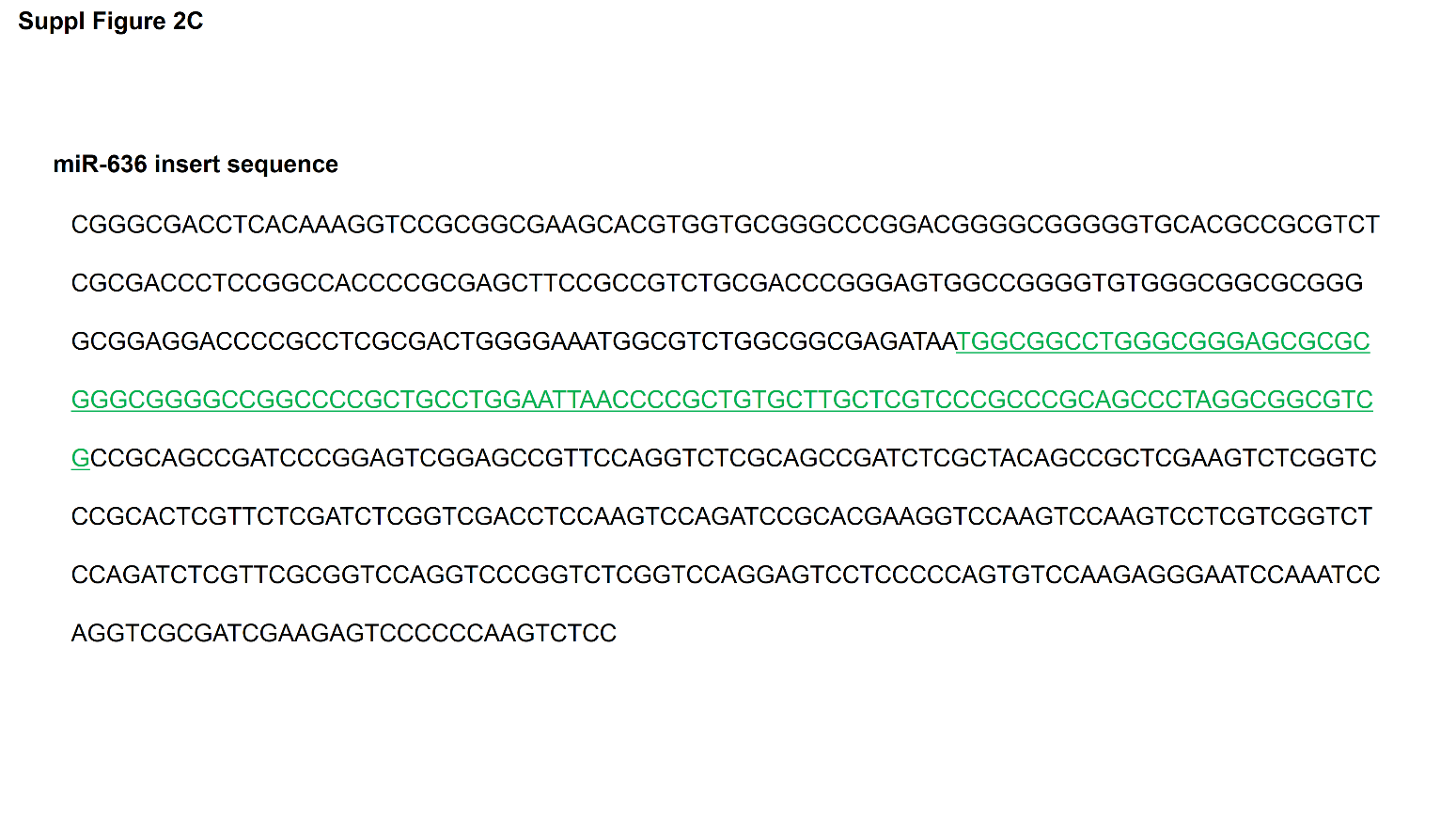
***

***Figure S2: Cloning of microRNAs in pcDNA3.1 backbone:*** *Schematic representation of cloning of miRNA-431 and miRNA-636 in pcDNA 3.1 construct****.*** *MicroRNA precursors for miR-431 and miR-636 were PCR amplified from genomic DNA and then cloned into pcDNA3.1 expression vector by standard PCR/restriction enzyme based cloning methods. Insert sequence of hsa-miR-431 and hsa-miR-636 with their respective stem-loop sequence is marked and underlined.*

*
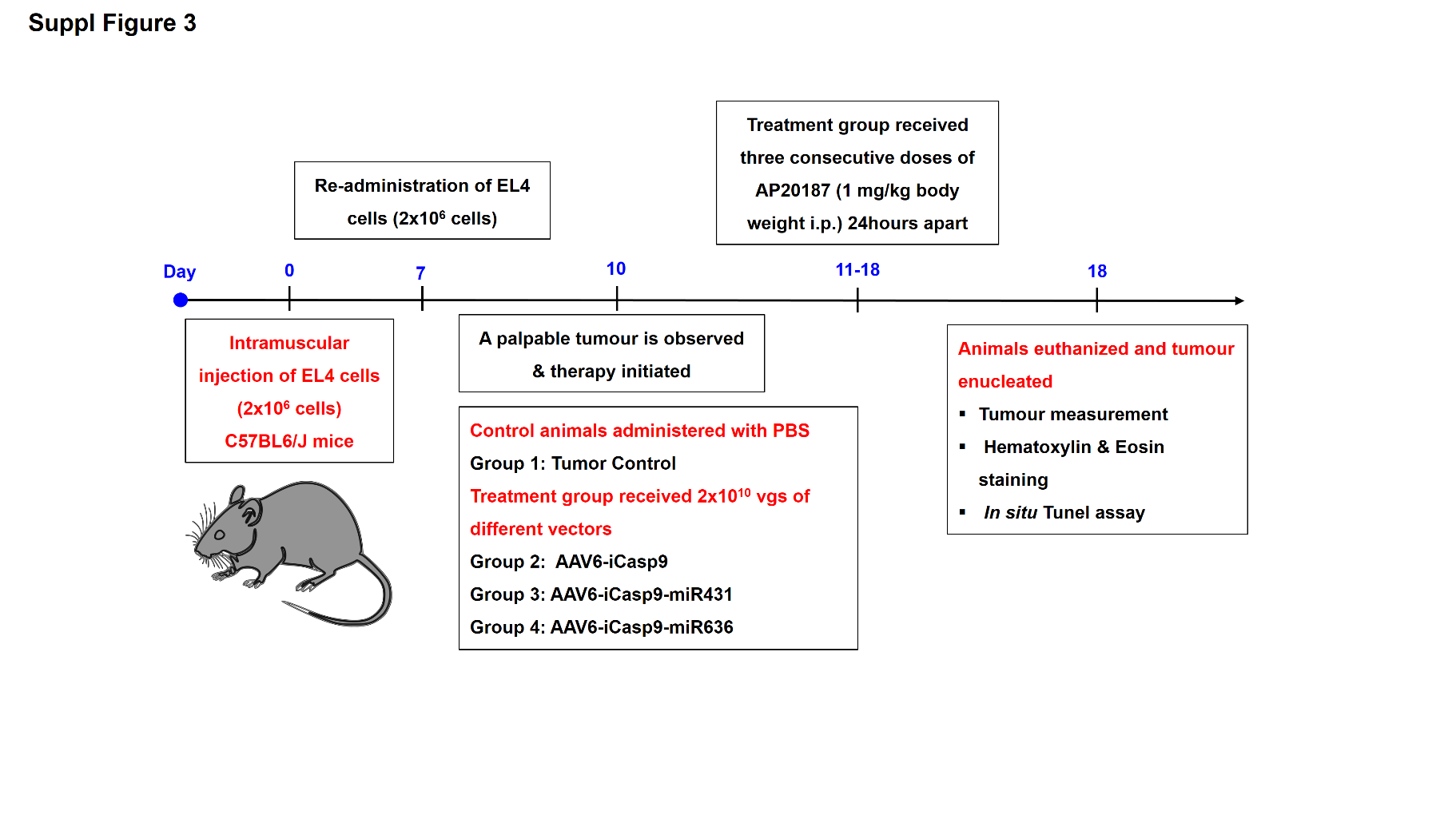
*

***Figure S3: Schematic outline for evaluation of suicide gene therapy with vectors packaged by either triple or quadruple method in a syngeneic model of murine lymphoma.***

| **Bioproject** | **Study accession No.** | **Bio-sample Accession No.** | **Run File Accession No.** | **Sample Description** |
| --- | --- | --- | --- | --- |
| **PRJNA564391** | **SRP220809** | SAMN12711515 | SRR10082853 | Control_A_triple transfection_Biological replicate 1 |
|  |  | SAMN12711516 | SRR10082854 | Control_B_triple transfection_Biological replicate 2 |
|  |  | SAMN12711517 | SRR10082855 | miR431_C_quadruple transfection_ Biological replicate 1 |
|  |  | SAMN12711518 | SRR10082856 | miR431_D_quadruple transfection_ Biological replicate 2 |
|  |  | SAMN12711519 | SRR10082857 | miR636_E_quadruple transfection_ Biological replicate 1 |
|  |  | SAMN12711520 | SRR10082858 | miR636_F_quadruple transfection_ Biological replicate 2 |

***Table S1: Sample submission details in the Sequence Read Archive (SRA).*** The transcriptome sequencing raw data obtained is submitted to SRA archive (https://submit.ncbi.nlm.nih.gov/subs/sra/SUB6272613) and their accession numbers are provided.
